# The Role of Aryl Hydrocarbon Receptor (AhR) in Brain Tumors

**DOI:** 10.3390/ijms21082863

**Published:** 2020-04-20

**Authors:** Maria L. Perepechaeva, Alevtina Y. Grishanova

**Affiliations:** Institute of Molecular Biology and Biophysics, Federal Research Center of Fundamental and Translational Medicine, Timakova Str. 2, 630117 Novosibirsk, Russia; agrish@niimbb.ru

**Keywords:** aryl hydrocarbon receptor, AhR, brain tumors, kynurenine pathway, therapeutic target

## Abstract

Primary brain tumors, both malignant and benign, are diagnosed in adults at an incidence rate of approximately 23 people per 100 thousand. The role of AhR in carcinogenesis has been a subject of debate, given that this protein may act as either an oncogenic protein or a tumor suppressor in different cell types and contexts. Lately, there is growing evidence that aryl hydrocarbon receptor (AhR) plays an important part in the development of brain tumors. The role of AhR in brain tumors is complicated, depending on the type of tumor, on ligands that activate AhR, and other features of the pathological process. In this review, we summarize current knowledge about AhR in relation to brain tumors and provide an overview of AhR’s potential as a therapeutic target.

## 1. Introduction

Primary brain tumors, both malignant and benign, belong to many types that occur in the brain parenchyma or meninges [1]. According to the Central Brain Tumor Registry of the United States, the average annual age-adjusted incidence rate of all malignant and non-malignant brain and other CNS tumors was 23.41 people per 100,000 [2].

The latest classification of central nervous system tumors according to the 2016 World Health Organization (WHO) guidelines is based both on the histological features of the tumor and on the most significant molecular genetic characteristics or chromosome aberrations and includes new morphomolecular nosological entities as compared to previous ones [3]. Nonetheless, according to the cellular composition, intracranial tumors can be roughly categorized into neuroepithelial (which develop directly from brain tissue), membranous (meningiomas, which develop from meningeal tissues), and glandular (which form from pituitary cells). Incidence, prevalence, and survival for brain tumors varies by histologic type, age at diagnosis, sex, and race/ethnicity. Meningiomas, pituitary tumors, and malignant gliomas are the most common types of brain tumors in adults [4].

**Figure 1:**
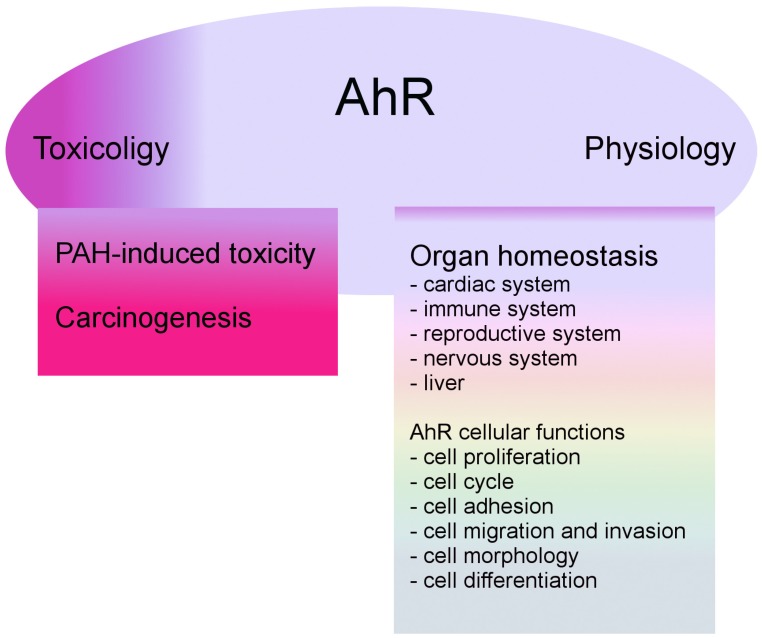
Integration of physiological and toxicological cellular functions by aryl hydrocarbon receptor (AhR). AhR plays an important role in toxicology, promoting the metabolism and elimination of toxic and carcinogenic compounds present in the environment. The AhR signaling pathway is also important in organ homeostasis and maintenance of critical cellular functions.

Traditionally regarded as a critical intermediate in the toxic and carcinogenic response to polycyclic aromatic hydrocarbons (PAHs) and dioxin (2,3,7,8-tetrachlorodibenzodioxin, TCDD), aryl hydrocarbon/dioxin receptor (AhR) is also an important regulator of cell physiology and organ homeostasis (Figure 1) [5]. For several decades, AhR has been studied in terms of toxicology and pharmacology. Furthermore, due to a substantial number of recent studies on its contribution to the proper functioning of the liver and cardiovascular, immune, and reproductive systems [6], AhR research has become a new, highly relevant field of study. Lately, there is growing evidence that AhR plays an important role in the initiation of benign and malignant brain tumors, including gliomas, meningiomas, medulloblastomas, and neuroblastomas [7].

## 2. Brain Tumors

The 2016 WHO Classification of Brain Tumors is based not only on the histological structure of a particular neoplasm, but also on its most significant molecular genetic characteristic or chromosome aberration [8]. This edition has added newly recognized neoplasms, and has deleted some entities, variants, and patterns that no longer have diagnostic and/or biological relevance.

Below we will discuss the role of AhR in the initiation of benign and malignant brain tumors, including gliomas, meningiomas, medulloblastomas and pituitary tumors.

The 2016 WHO edition presents a major restructuring of the diffuse gliomas, medulloblastomas, and other embryonal tumors. It incorporates new entities that are defined by both histology and molecular features. Tumor diagnoses should consist of a histopathological name followed by the genetic features (isocitrate dehydrogenase (IDH) status). For those entities with more than one genetic determinant, the multiple necessary molecular features are included in the name (1/19q codeletion and other genetic parameters).

Gliomas account for ~75% of malignant primary brain tumors, the vast majority of which are glioblastomas [1]. The 2016 WHO Classification presents a restructuring of diffuse astrocytic and oligodendroglial tumors and includes:

Diffuse astrocytotoma, IDH-wildtype;

Diffuse astrocytotoma, IDH-mutant;

Diffuse astrocytotoma, NOS (not otherwise specified);

Anaplasic astrocytotoma, IDH-wildtype;

Anaplasic astrocytotoma, IDH-mutant;

Anaplasic astrocytotoma, NOS;

Glioblastoma, IDH-wildtype;

Glioblastoma, IDH-mutant;

Glioblastoma, NOS;

Diffuse midline glioma, H3 K27M-mutant;

Oligodendroglioma, IDH-mutant and 1/19q codeled

Oligodendroglioma, NOS;

Anaplastic oligodendroglioma, IDH-mutant and 1/19q codeled

Anaplastic oligodendroglioma, NOS;

Oligoastrocytoma, NOS;

Anaplastic oligoastrocytoma, NOS.

Other notable changes of the 2016 WHO Classification include the addition of brain invasion as a criterion for atypical meningioma. Meningiomas are tumors that arise from arachnoid cells (meningothelial cells). They are a common primary intracranial tumor in adults, accounting for 37% of primary brain tumors in the United States [9]. Meningiomas are usually slow-growing tumors, although there are more aggressive but less common subtypes. Meningiomas are more common among women than men. Approximately 80% of meningiomas are benign tumors, but 15–20% are atypical (class II according to WHO) and anaplastic (class III according to WHO), which are characterized by a high recurrence rate and resistance to standard treatments [9,10]. Current treatments of malignant meningiomas include surgical resection followed by radiation therapy [11]. Chemotherapy is rarely used in clinical practice [10]. Currently, there are no FDA-approved chemotherapeutic agents that are effective against meningiomas, although hydroxyurea has a small effect [12]. Pituitary tumors, glandular tumors of the brain, constitute 10–25% of intracranial neoplasms [13]. Most pituitary adenomas are considered benign. Some adenomas discovered by chance on MRI are not associated with clinical symptoms [13], and others may have a wide range of clinical manifestations [14]. The anterior pituitary gland consists of several types of hormone-producing cells, from which a heterogeneous group of neoplasm-producing hormones can form that reflect their origin, with the corresponding systemic effects [13]. The accumulation of genetic mutations causes such oncogenic changes as steady proliferation, invasion, angiogenesis, evasion of growth inhibition, and resistance to cell death. Nevertheless, in general, mutations that control oncogenesis are rare in pituitary tumors. Non-mutational sources of change in the gene expression of pituitary tumors are being studied [15]. Medulloblastomas are a primary brain tumor in children, deriving from abnormally proliferating precursors of cerebellar neurons. The 2016 WHO Classification presents a restructuring of medulloblastomas and other embryonal tumors, medulloblastomas, WNT-activated, medulloblastomas, SHH-activated, and embryonal tumors with multilayered rosettes, C19MC-altered [8].

## 3. AhR Biology

### 3.1. AhR Regulation

AhR is a ligand-activated transcription factor belonging to the bHLH/PAS (basic helix-loop-helix/Per-Arnt-Sim) family of nuclear receptors. Members of this family sense a variety of extra- and intracellular signals, including endogenous or exogenous chemicals [16]. These signals are converted into a cellular response as a result of a signal transduction process, the molecular components of which form the corresponding signaling pathway.

**Figure 2:**
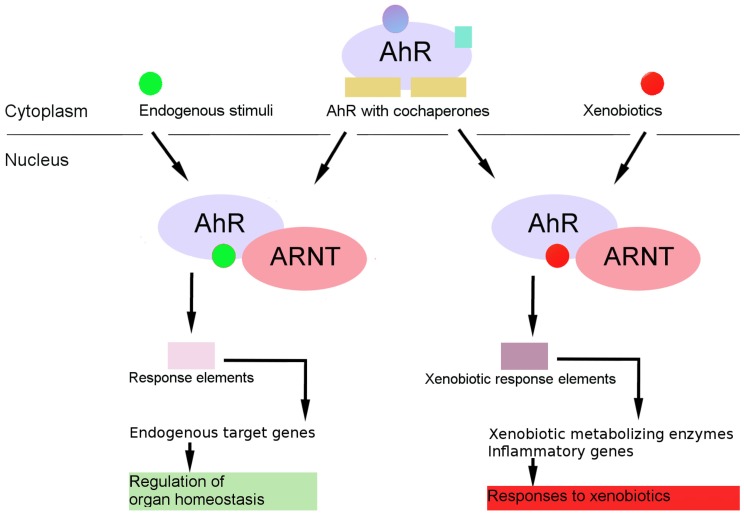
The AhR genomic pathway. AhR resides in cytosol in a complex of chaperones and other proteins. Upon ligand (xenobiotics or endobiotics) binding, AhR changes conformation. This allows the translocation of the ligand–receptor complex into the nucleus and the dissociation of the receptor complex. In the nucleus, the complex dimerizes with its partner ARNT (or other partners from other signaling pathways) and this complex binds to DNA in certain elements and recruits transcription cofactors. Target genes are transcribed, leading to cell-specific transcriptome changes.

The activation of nuclear receptors is carried out in various ways. In particular, the trigger may be the binding of a ligand to an inactive receptor; this is the activation mechanism that is considered classic for AhR (Figure 2). In the absence of a ligand, AhR is in the cytoplasm in complex with two Hsp90 (heat shock protein 90) molecules, cochaperone p23, and the XAP2 protein, also known as ARA9 or AIP [17]. Hsp90 interacts with the ligand-binding PAS-B and bHLH domains of AhR. After the binding of AhR to the ligand, cochaperones are released, and the AhR–ligand complex is translocated into the nucleus, where it dimerizes with the ARNT partner protein, which also belongs to the bHLH/PAS family [6,18]. The AhR–ARNT heterodimer binds to a xenobiotic response element (XRE; one of xenobiotic-sensitive elements, consensus sequence 5′-TNGCGTG-3′) in DNA, thereby enhancing appropriate functions. Signal transmission from an enhancer to a promoter is associated with the activity of AhR TA domains and various transcription coactivators. As a result, the transcription of target genes starts; these genes encode xenobiotic biotransformation enzymes as well as murine epiregulin, ecto-ATP, δ-aminolevulinic acid synthase, prostaglandin endoperoxide H synthase 2, MDR1, BRCP, AhRR, and p27kip1 [19,20,21].

At the molecular level, AhR regulates the expression of a wide range of physiologically relevant genes [22]. The regulation is carried out either via traditional transcription-dependent mechanisms or through processes involving genomic insulators, chromatin dynamics, and transcription of mobile genetic elements. Additionally, AhR is closely related to epigenetics, not only in terms of target gene expression but also in terms of its own regulation by the promoter methylation.

Endogenous physiological ligands of AhR were characterized by means of heme degradation products (bilirubin and biliverdin) [23], metabolites of arachidonic acid (LXA4) [6,24], tetrapyrroles [6], and tryptophan metabolites such as kynurenine [25] and kynurenic acid [26]. The discovery of the endogenous ligands suggests that AhR might function in normal physiological processes [27].

At the cellular level, AhR engages in functional interactions with signaling pathways that regulate cell proliferation and the cell cycle, cell morphology, cell adhesion, and cell migration.

The AhR signaling cascade can interact with signal transduction pathways of other transcription factors, such as nuclear factor NF-κB, which regulates apoptosis, development, immune responses, and the stress response [28], and HIF-1α (a hypoxia-induced factor) which is involved in the regulation of immunity, circadian rhythms, and tumor progression [29,30,31]. There is cross-talk between the AhR signaling pathway and Nrf2 (nuclear factor erythroid 2-related factor 2), the main regulator of the endogenous antioxidant defense system of the human body [32,33,34,35]. Perhaps there is a relation between AhR and the Wnt signaling pathway, which triggers a cascade of reactions that regulate normal development of the embryo and participate in cell homeostasis throughout the lifespan in most tissues and organs [36].

It is reasonable to assume that deregulation of the physiological functions of AhR may play a causal role in (or at least contribute to) human disease [37].

### 3.2. AhR and the Nervous System

The functions of AhR in the nervous system have not been sufficiently studied to date. Research on invertebrates in which AhR orthologs are expressed in neural cells shows that these orthologs differ from AhR of vertebrates: they do not bind to the “classic” vertebrate AhR ligand, dioxin [38], although AhR signaling pathways of vertebrates and invertebrates have common features. It is likely that the ability to bind to PAH-like compounds has been acquired by AhR at later stages of evolution owing to mutations in the PAS domain [39].

As for vertebrates, it is known that AhR is expressed and this expression increases as neural progenitor cells for the hippocampus and cerebellum, as well as non-neural brain cells, astrocytes, and endotheliocytes, and the blood–brain barrier, are formed in the mouse body [39]. Brain expression of AhR oscillates regularly in the suprachiasmatic nucleus of the mouse brain, the hypothalamic region that controls circadian rhythms [40]. Moreover, the expression of AhR in the nervous system of rodents depends not only on internal stimuli but also on external ones, such as traumatic brain injury, exposure to xenobiotics, and endocrine disorders [39].

A neuroactive metabolite tryptophan, called kynurenic acid, is involved in the pathogenesis of mental illness and is an AhR ligand [41]. An increase in the kynurenine/kynurenic acid ratio in the brain is associated with the symptoms of chronic fatigue syndrome, depressive disorders, and other psychiatric conditions [42]. In this regard, it is proposed to increase the level of kynurenic acid in brain tissues for therapeutic purposes, but the question arises whether activation of AhR by kynurenic acid will increase the risk of cancer [41]. On the other hand, kynurenine is an AhR ligand too [25] but additionally mediates processes in the pathogenesis of mental disorders.

In the tissues of the central nervous system, AhR can be activated by tryptophan molecules produced by intestinal bacteria. Tryptophan can cross the blood–brain barrier and activate AhR, which, apparently with the participation of interferons, inhibits the NF-κB signaling pathway and reduces the production of proinflammatory cytokines [43]. The notion that AhR “mediates” the interactions between the host and microbiota may be supported by the fact that AhR expression is low in bacteria-free mice [44].

## 4. AhR in Neurological Cancer

Emerging evidence indicates an oncogenic role of AhR in the initiation, promotion, progression, invasiveness, and metastasis of cancer.

AhR is overexpressed in various types of tumors and tumor cell lines, suggesting that AhR is activated constitutively in tumors and facilitates their growth [45]. The activation of AhR often contributes to clonogenicity and invasiveness of cancer cells [46]. AhR affects various types of immune cells and is capable of inducing immunosuppression, and some types of cancer avoid recognition by immune cells via this mechanism [45].

Lately, there is growing evidence that AhR plays an important part in the initiation of benign and malignant brain tumors, including gliomas, meningiomas, and medulloblastomas [7]. The involvement of AhR in brain cancer is complicated, depending on the type of cancer, on ligands that activate AhR, and other features of the pathological process.

### 4.1. AhR in Gliomas

For glioblastomas, the high activity of TGF-β/Smad signal transduction pathways is typical and associated with a poor prognosis. Transforming growth factor (TGF)-β is the central mediator of the malignant phenotype of these tumors and promotes invasiveness and angiogenesis while maintaining cancer stem cells and causing strong immunosuppression [46,47].

It is known that TGF-β plays the role of AhR in signaling, and AhR is expressed in glioma cells in vitro (malignant glioma cell lines U87MG and T98G) and is detectable in human gliomas and glioblastomas (anaplastic astrocytomas WHO grade III and primary and recurrent glioblastomas grade IV [48]) in vivo by immunohistochemistry with predominantly nuclear staining [46,49]. AhR activity positively controls the levels of TGF-β1, TGF-β2, and latent TGF-β–binding protein 1 in malignant glioma cells [46].

It has been shown on glioma cell lines that AhR promotes the development of a malignant phenotype of glioma cells at the level of proliferation, clonogenicity, and invasiveness: these properties weaken during exposure to a low-molecular-weight inhibitor of AhR (CH-223191) or after an AhR knockout. The latter has a less pronounced effect than CH-223191 does; therefore, it is possible that different AhR activities are differentially sensitive to inhibition as a result of coregulation with many other pathways and cofactors [7,46,50,51].

Thus, the pathogenesis of gliomas may include altered regulation of AhR, which may be a promising target for the treatment of malignant human gliomas and other diseases associated with a pathological activity of TGF-β [46,47,52,53].

It is believed that gliomas form as a result of an interaction between genetic factors and environmental factors. As far as the biomedical literature is concerned, the only well-documented risk factor of glioma is ionizing radiation. Several genetic variants have been identified that are associated with an increased risk of glioma (glioma grade I-IV, WHO 2007 [48]), but its etiology is not fully clear [54].

Exposure to polycyclic aromatic hydrocarbons (PAHs) is known to increase the risk of some cancers, and there are some reasons to believe that these include brain tumors. Activation of AhR under the influence of PAHs leads to cell cycle disturbances, a decrease in the DNA replication ability, and inhibition of cell proliferation. Because AhR is involved in the response to PAHs, genetic variants of AhR can increase the risk of glioma after exposure to PAHs. Two of the six AhR polymorphisms that have been studied, rs2066853 and rs2158041, are significantly associated with glioma risk and levels of PAH–DNA adducts in glioma tissue [54].

### 4.2. AhR in Meningiomas

A study on the AhR-dependent signaling pathway in human meningiomas showed that its components are enhanced in these tumors. The level of AhR mRNA is higher in meningioma tissue than in the corresponding normal tissue and higher in more malignant tumors, and AhR is more abundantly localized in the nucleus, thus indicating its activation [49,55]. Besides, in meningiomas, compared with the normal tissue and with increasing tumor malignancy (grade I, II, III [48]), the regulation of genes of AhR-dependent signal transduction, e.g., *ARNT*, aldehyde dehydrogenase 1A3, and *CYP1A1*, is enhanced [55].

The AhR-dependent signal transduction pathway is known to include the inhibition of apoptosis in cancer cells. This notion is confirmed by upregulation of cyclophilin D in all classes of meningiomas, and this protein participates in the formation of high-permeability pores in mitochondria during apoptosis, and a decrease in the amount of the c-Fos protein regulated by AhR. In contrast to its normal function of a proto-oncogene, in some tumors, *c-Fos* acts as a tumor suppressor and functions during apoptosis [56].

### 4.3. AhR in the Pituitary Adenoma

Recently, the AhR signaling cascade was identified as a key pathway suppressed in pituitary adenomas, and there is conflicting evidence on both the tumor-suppressive and oncogenic role of AhR depending on the specificity of cells and a tissue context. AhR appears to be expressed in all pituitary cell lines whose tissues of origin are sensitive to AhR agonists via the classical signal transduction pathway [57].

AhR can negatively affect the endocrine system and the hypothalamic–pituitary–gonadal axis: for example, during treatment of mice with TCDD during early pregnancy, AhR dependently reduces the expression of gonadotropins in the pituitary gland of cubs, leading to an impairment of reproductive function [58]. Evidence is now reported of the equally important function of AhR in the regulation of the growth, proliferation, and apoptosis of pituitary cells and in the formation or progression of human pituitary adenomas [57].

Research conducted at sites of technological disasters or under unfavorable environmental conditions with dioxin emissions has shown that in one study (in Seveso, Italy), there were no significant changes in the incidence of pituitary adenoma [59], and in another (in Messine, Italy), there was an increased incidence of acromegaly as a result of a tumor, which was associated with *AhR* polymorphic variants rs2066853 and rs4986826 [60].

Most studies on the genetic predisposition to pituitary adenoma have dealt with the partner protein of AhR (AIP), which, like AhR, is expressed in the normal human pituitary gland. Approximately 40% of patients with pituitary adenoma have AIP mutations, which lead to familial predisposition to this disease. Because mutations inactivate AIP, it is itself regarded as a tumor suppressor gene for the pituitary tumor [61].

By immunohistochemical methods, it was shown that the AhR protein is less abundant in samples of pituitary adenomas compared to samples of a normal pituitary gland, and its amount correlates with AIP expression. Thus, the participation of AIP in the cytoplasmic stabilization of AhR in the human pituitary gland is confirmed, and accordingly, so is the destabilization of AhR during AIP deficiency, which can occur in pituitary adenomas carrying mutant inactive AIP [61].

In contrast, AhR appears to be activated in nonsecreting adenomas overexpressing AIP, which were found in patients in Seveso [59]. This finding points to a different function of AhR in pituitary cells.

AhR can affect the cell cycle in the absence of an exogenous ligand, regardless of its role as a xenobiotic receptor. In the rat GH3 cell line, the most popular in vitro model of secreting tumors, overexpression of AhR (either in the absence or presence of the AhR ligand benzo[α]pyrene) has been shown to reduce cell proliferation, and this effect disappears after an AhR knockdown.

AhR has been demonstrated to inhibit the cell cycle by interacting with the Rb1 protein, thereby resulting in the inhibition of E2F-mediated transcriptional activity. Changes in the expression of cell cycle regulators CDK–CDKN were also found in pituitary adenomas, with a general increase in the expression of cyclin and CDK genes and CDKN underexpression as compared with the normal cells [62]. This phenomenon can also be one of the mechanisms by which excessive AhR expression reduces cell proliferation and inhibits cell cycle progression [62].

### 4.4. AhR in Embryonal Tumors

Medulloblastoma, a primary brain tumor in children, derives from abnormally proliferating precursors of cerebellar neurons in which AhR is overexpressed. Observations that impairment of AhR function disturbs the regulation of the cell cycle of cerebellar neuronal precursors suggest that AhR promotes medulloblastoma growth [63]. In an immortalized cell line of medulloblastoma (DAOY cells) with a knockdown of AhR, in which the AhR protein level is decreased by 70% as compared to wild-type DAOY cells, cell cycle disturbances, decreased DNA synthesis, and decreased proliferation are observed. AhR addback restored the cell proliferative activity. That is, AhR promotes the proliferation of medulloblastoma cells and should be regarded as a potential therapeutic target in this disease [63].

Neuroblastoma, a tumor originating from primitive cells from the neural crest of the autonomic nervous system, is one of the most common extracranial cancers among children and infants [64]. Molecular defects of differentiation are some of its etiologies, and dysregulation of miR-124 is crucial. The latter is a highly conserved microRNA that is specifically expressed in the nervous system. It was found that in the absence of miR-124 in SK-N-SH neuroblastoma cells, AhR protein expression increases while cell proliferation slows and the cell cycle is blocked [65].

## 5. AhR and the Kynurenine Pathway (KP) of Tryptophan Metabolism in Brain Tumors

**Figure 3:**
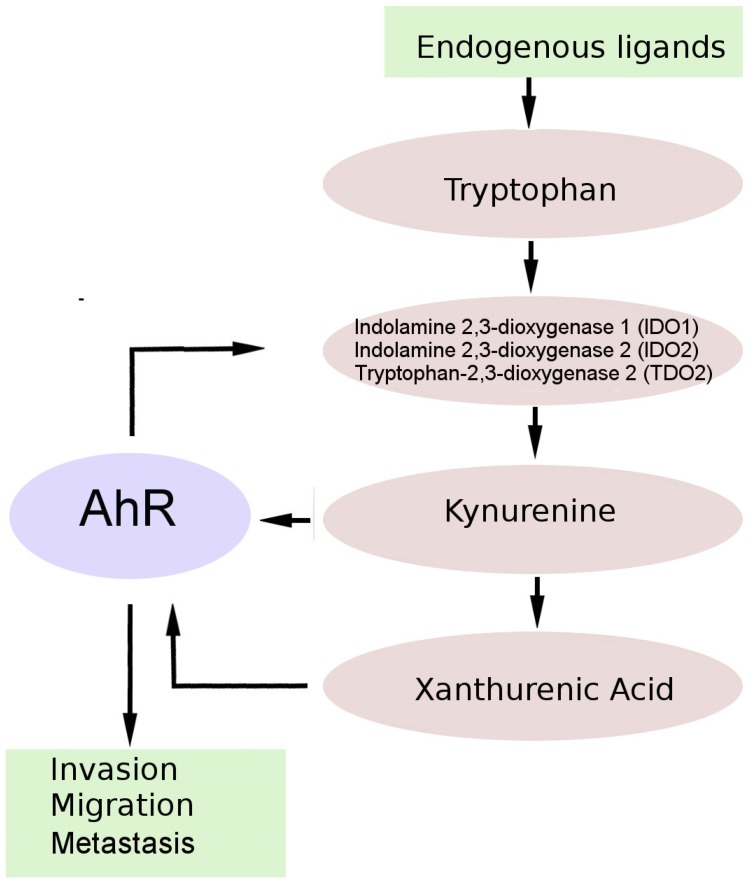
An endogenous tumor-promoting ligand of AhR in brain tumor cells. AHR in tumor cells is chronically activated by kynurenine pathway metabolites, including kinurenine and xanthurenic acid. AHR activation leads to increased regulation of genes associated with tumor cell invasion, migration, and metastasis. AHR also enhances the expression of IDO and/or TDO in malignant cells, resulting in continuous production of endogenous AHR ligands.

There is growing evidence supporting the role of changes in tryptophan metabolism (via the kynurenine pathway, KP) in the pathogenesis of primary brain tumors [66]. Tryptophan is an essential amino acid necessary for protein biosynthesis, and more than 95% of the tryptophan that is not incorporated into proteins is metabolized via the KP. Normally, this pathway generates active metabolites and is the main source of NAD^+^. By contrast, in cancer patients, the KP is deregulated, resulting in local tryptophan depletion and overproduction of active metabolites, which leads to the formation of an immunosuppressive tumor microenvironment (Figure 3) [49,67].

The initial KP rate-limiting stage, in which tryptophan is converted to N-formylkynurenine, is carried out by three enzymes: indolamine 2,3-dioxygenase 1 (IDO1), indolamine 2,3-dioxygenase 2 (IDO2), and tryptophan-2,3-dioxygenase (TDO2). N-formylkynurenine is converted to kynurenine, the central metabolite of the KP, and can be further metabolized into several neuroactive metabolites: kynurenic acid, 3-hydroxykynurenine, and quinolinic acid [49,68].

It is well known that both primary and secondary brain tumors overexpress IDO1 and to some extent IDO2 [69,70].

Kynurenine suppresses T-cell proliferation, which inversely correlates with the formation of kynurenine by TDO2, which is thought to be the predominant rate-limiting enzyme in the KP of gliomas. Sections of human glioma with high TDO2 expression show a decrease in infiltration by LCA^+^ and CD8^+^ immune cells as compared with cells underexpressing TDO2, suggesting that the formation of kynurenine by TDO2 can suppress the antitumor immune response. A knockdown of this enzyme causes lysis of glioma cells by peripheral blood mononuclear cells [71]. In vivo experiments on immunocompetent mice reveal that tumors expressing TDO2 grow faster and have a higher proliferation index than TDO2-deficient tumors do [71].

Kynurenine is a pluripotent mediator and a key intermediate for the synthesis of many KP metabolites that are involved in inflammation, immune modulation, and neurological reactions [67]. On the other hand, kynurenine and kynurenic acid are AhR ligands, and the discovery that KP metabolites are AhR ligands suggests that, among other things, the KP uses the AhR-dependent signal transduction pathway to increase the survival and motility of tumor cells [49,56]. In addition to the formation of AhR agonists kynurenine and kynurenic acid, tryptophan is the source of several other AhR high-affinity ligands, including FICZ and ITE [67].

The TDO2–kynurenine–AhR pathway is active in human brain tumors and is associated with malignant progression and poor survival. TDO2 is overexpressed in human glioma cells, and this phenomenon correlates with the production of kynurenine and with the expression of AhR target genes, thus indicating the production of a sufficiently large amount of kynurenine for AhR activation [71]. The role of AhR in tumor growth is confirmed by the slower growth of TDO2-expressing tumors in AhR-deficient mice and the abrogation of clonogenic survival in response to kynurenine in AhR knockdown glioma cells [71].

There is evidence that the TDO2–kynurenine–AhR signaling pathway is not specific for brain tumors but rather is a common feature of cancerous tumors [71]. Note that a kynurenine concentration sufficient to activate AhR is generated in response to inflammation, and a substantial number of malignant tumors arise from sites of chronic infection and inflammation [71].

## 6. The Therapeutic Potential of AhR Targeting

Despite advances in cancer therapy and a reduction in the mortality rate of this group of diseases in general, the mortality rate of patients with brain tumors has remained relatively constant in recent decades. The poor prognosis and a lack of effective treatments of malignant brain tumors underscore the need to develop new therapeutic strategies against these diseases [49].

Tryptophan metabolism pathways and the AhR signaling pathway, which is activated in response to kynurenine, and some other components of the KP, are dysfunctional in gliomas and meningiomas [55,70,72] as well as in a number of other solid tumors [73,74].

Several dozen drugs targeting nuclear receptors have already been approved for use in clinical practice, but there are no compounds targeting AhR so far [7]. Currently, approaches are being devised to “intercept” the nodal points of the IDO/TDO–kynurenine–AhR enzymatic/signaling cascade. IDO1/TDO2 inhibition should prevent the formation of AhR ligand kynurenine and activation of AhR [67], and prevent kynurenine-mediated immunosuppression and neurotoxicity induced by quinolinic acid [49].

IDO1/TDO2 inhibitors suppress tumor formation in animal models and are currently being evaluated in clinical trials against a wide range of cancers, including melanoma, glioblastoma, non–small cell lung cancer, and pancreatic and breast cancers [67,71,75]. First-generation IDO1 inhibitors have not manifested a significant antitumor activity in patients; second-generation IDO1 inhibitors are more effective, but overall success is modest, either as monotherapy or in combination with immune control point inhibitors [67]. Unfortunately, the IDO1 inhibitor epacadostat in combination with the anti-PD-1 antibody pembrolizumab in a phase 3 clinical trial in patients with unresectable stage III or IV melanoma did not improve progression-free survival or overall survival compared with placebo plus pembrolizumab [76]

Cancer immunotherapies can yield poor results, and one of the reasons is that activation of AhR by kynurenine leads to the formation of immunotolerant dendritic and regulatory T cells, which contribute to the creation of an immunosuppressive tumor microenvironment [67]. Research points to the central role of AhR in the induction of tolerogenic immune responses. AhR regulates and controls the functions of dendritic cells, macrophages, natural killers, Innate lymphoid cells (ILCs), T helper 17 (Th17) lymphocytes, Th22 lymphocytes, and regulatory T cells [67,77].

The development of antitumor therapy by direct inhibition of AhR is in its infancy and requires a more complete understanding of the involvement of AhR in cancer initiation/progression and in the functioning of the immune system [67]. Nonetheless, the use of small-molecule compounds to modulate IDO1/2, TDO2, and AhR in glioma and meningioma cell lines has already proven that in contrast to enzyme inhibition, AhR antagonists markedly reduce tumor cell viability, i.e., AhR may be a therapeutic target in these types of cancer. Inhibition of enzymes that limit the rate of KP has not changed the viability of cancer cells in vitro, probably because the antitumor impact of KP inhibition is associated with triggering of the immune system, and this factor is absent in the in vitro system [49].

In addition, there may exist other endogenous AhR ligands that switch AhR on in the absence of kynurenine [49]. Therefore, inhibition of AhR by means of synthetic modulators is a promising approach because it will attenuate the immunosuppressive effect of any AhR ligands.

The immune system begins to deteriorate during aging, and given that the average age of patients with glioblastomas and malignant meningiomas is ~65 years [2], cytotoxic agents can further suppress immunity. Consequently, such patients should benefit from drugs that have other mechanisms of action, e.g., AhR antagonists [49].

On the other hand, the immune response induced by AhR ligands depends on the type of ligand, the type of tumor, and characteristics of the pathological process. Although the dominant interpretation of the immunomodulatory effect of activated AhR is immunosuppression, in pituitary adenomas and neuroblastomas, on the contrary, activation of the antitumor immune response through AhR-dependent mechanisms is observed.

When designing selective AhR modulators, researchers should take into account possible effects on the classic AhR signaling pathway, depending on ligand-selective binding of the AhR complex to nontraditional sequences of xenobiotic response elements or interaction with various subgroups of coactivators [78]. There are complex relations among IDO/TDO, kynurenine, and AhR, which are disrupted by the progression of cancer [67]. Apparently, in different cases, AhR antagonists or agonists can exert either a pro- or antitumor influence, modulating immune responses and acting directly on cancer cells. Therefore, additional theoretical knowledge about these relations is required, which will allow for the correct design of pharmacological agents aimed at AhR and thorough testing of such agents.
